# Q Fever in Africa: A Systematic Review of Prevalence, Zoonotic Potential and Disease Burden

**DOI:** 10.1002/puh2.70323

**Published:** 2026-08-05

**Authors:** Linda Ama Owusuaa Amoah, Richard Kwamena Abbiw, Naa Awula Narkie Nartey, Becky Ewurama Tetteh, Elizabeth Bugase

**Affiliations:** ^1^ Council for Scientific and Industrial Research‐Animal Research Institute Achimota, Accra Ghana; ^2^ West African Centre for Cell Biology of Infectious Pathogens (WACCBIP) University of Ghana, Legon Accra Ghana; ^3^ African Centre for Climate Resilience and Adaptation Accra Ghana

**Keywords:** Africa, Coxiella burnetii, coxiellosis, One Health, prevalence, query fever/Q fever, reservoir hosts

## Abstract

**Introduction:**

Q fever, caused by *Coxiella burnetii*, is increasingly recognised in Africa, though its burden remains underestimated due to limited surveillance and underreporting. Reliable prevalence and epidemiological data are crucial for understanding the disease's distribution, identifying high‐risk populations and developing effective control strategies, necessitating this systematic review.

**Methods:**

The review protocol is registered in prospectively registered systematic reviews (PROSPERO) (ID: CRD42025625366). Research questions and inclusion/exclusion criteria were developed using the condition, context and population (CoCoPop) framework. Relevant articles from 2004 to 2024 were sourced from PubMed, Web of Science, Science Direct and Google Scholar. Data were extracted using the Kobo Toolbox, and the Joanna Briggs Institute (JBI) Critical Appraisal Checklist was used to assess risk of bias. Descriptive analyses were conducted using R, and findings are presented in narrative form, with supporting tables and figures.

**Results:**

The review identified 136 articles which focused on domestic animals (66.9%), humans (33.1%), arthropods (25.0%) and wildlife (10.3%). Mean molecular prevalence varied among hosts as follows: goats (7.28% ± 8.84%), sheep (9.88% ± 11.95%) and cattle (8.20% ± 9.73%). Seroprevalence rates were higher, with camels showing the highest at 31.68% (standard deviation [SD] = 26.14). None of the studies addressed the economic burden of Q fever on human health and livestock production.

**Conclusion:**

This review highlights the prevalence of Q fever across Africa. It suggests the persistence of the disease is driven by a complex interplay among diverse host species, with livestock as the primary source of human infections. Findings emphasise the need for a One Health approach in all surveillance and for assessing the economic impacts of *C. burnetii* to effectively target and control the disease.

## Graphical abstract

Q fever is widespread across Africa, with livestock as the primary source of human infections. Although some prevalence data exist, its economic burden is still lacking. The findings underscore the need for a One Health approach that integrates surveillance, economic assessment and control to reduce the disease burden.

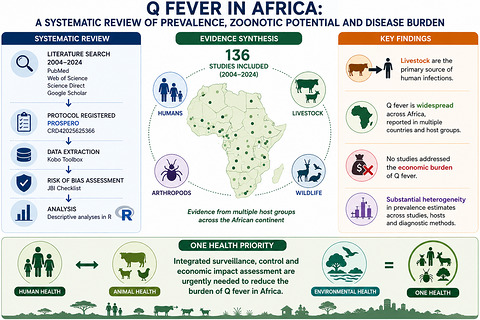

## 1 Introduction

Query fever (Q fever) is a zoonotic bacterial disease with worldwide distribution except for New Zealand and Antarctica [1, 2, 3]. This highly infectious disease is caused by *Coxiella burnetii*, an intracellular, gram‐negative coccobacillus bacterium. This pathogen is highly resistant to both low and high pH levels and ultraviolet radiation and is efficient in forming a spore‐like small cell variant (SCV), which is environmentally resistant and can serve as inocula for delayed outbreaks [4, 5]. Q fever is also a common cause of fever in intertropical areas, with disease prevalence varying from country to country due to epidemiological differences and reporting practices [6, 7].

*C. burnetii* infects a wide variety of mammals and non‐mammals, including reptiles, birds and amphibians [8, 9]. However, the primary reservoir hosts are ruminant livestock (cattle, sheep and goats), although companion animals, such as dogs and cats, and arthropods, like ticks, lice and fleas, can serve as sources of infection [10, 11, 12]. Although birds are identified as potential reservoirs of *C. burnetii*, their role in the distribution and maintenance of the pathogen has not been completely verified [13]. Once an animal is infected, *C. burnetii* can localise in various tissues, including the mammary glands, supramammary lymph nodes, placenta, amniotic fluid, urine, faeces and uterus [14].

*C. burnetii* infections in humans can manifest acutely or chronically, with an estimated 40% of infected individuals developing symptomatic disease, typically presenting with self‐limiting and non‐specific influenza‐like symptoms [15]. The acute disease can be severe, manifesting as hepatitis or pneumonia and leading to hospitalisation or death, especially in cases of concomitant infections [16]. Chronic Q fever poses a significant threat to life, usually manifesting as chronic endocarditis [2, 17]. Treatment also differs between these stages of infection [18]. Transmission occurs mainly through contaminated aerosols produced during abortion, birth and the handling of birth products or from contact with infected domestic animals or unpasteurised milk [2, 18].

There is evidence indicating that Q fever imposes a persistent, long‐term burden on both humans and society, resulting in direct and indirect socioeconomic costs [14, 19]. *C. burnetii*, a known abortifacient, has significant economic impacts on livestock [20, 21]. Infected animals may experience reproductive disorders, such as abortions, retained placenta, stillbirths, premature deliveries or the birth of weak offspring, as well as a reduced conception rate or metritis [22, 23, 24]. Unquestionably, the abortion‐related losses in livestock production significantly impact the poorest communities worldwide, where millions depend on livestock for their economic livelihoods, food security, health and overall well‐being [25, 26]. The economic repercussions of the Netherlands Q fever outbreak (2007–2010) were estimated to be approximately €0.307 billion [27].

Several studies across the African continent have reported the prevalence of *C. burnetii* infections in humans, as well as in both domestic and wild animals [28, 29, 30, 31]. However, these studies are often small‐scale with uneven geographic coverage, leaving major knowledge gaps on the epidemiology and zoonotic potential of the disease on the continent. In nations where Q fever surveillance is virtually non‐existent, information about the disease's occurrence and its zoonotic implications is very limited. This lack of information hinders stakeholders’ ability to monitor and respond effectively to potential outbreaks and the emergence of the disease.

The primary aim of this review was to systematically examine the prevalence of Q fever in humans, domestic animals, wildlife and arthropods across Africa from 2004 to 2024 and also evaluate its economic impact by synthesising existing knowledge. Accurate and comprehensive data on the prevalence of Q fever in Africa are crucial for effective policymaking, targeted interventions, early detection and monitoring. Given that this information is vital for safeguarding global health, food security and improving healthcare [15, 32], conducting this systematic review is necessary. It also addresses a critical knowledge gap and identifies potential avenues for future research and interventions, especially in resource‐limited settings.

## 2 Methods

### 2.1 Review Protocol

The protocol for this systematic review is registered in the international database of prospectively registered systematic reviews (PROSPERO) with the following ID: CRD42025625366 (https://www.crd.york.ac.uk/PROSPERO/view/CRD42025625366). This study was conducted according to the Preferred Reporting Items for Systematic Review and Meta‐Analysis (PRISMA) guidelines to ensure all the elements required for a systematic review were available.

### 2.2 Search Strategy

We used the CoCoPop (condition, context and population) framework to formulate research questions that guided our search strategy and inclusion and exclusion criteria. The condition (Co) of focus was Q fever or *C. burnetii*, with the context (Co) referring to Africa, which we further divided into West, East, Central, South and North Africa. Our population (Pop) of interest included humans, domestic animals, including sheep, goats, cattle, camels and dogs, wildlife and arthropods (including ticks and fleas). To identify relevant articles within the specified timeframe (January 2004–December 2024), we searched published English literature using three scientific databases (PubMed, Web of Science and Science Direct) and a website (Google Scholar). To ensure the quality of our findings, we excluded all unpublished reports or theses from consideration.

We also reviewed related articles identified during the search of selected papers, as these served as Supporting Information. To ensure comprehensive results, we used the following keyword categories for our searches: ‘Q fever’, ‘*C. burnetii’*, ‘*C. burnetii* infections’, ‘query fever’, ‘*C. burnetii* vector‐borne disease’, ‘prevalence of Q fever’, ‘economic impact of Q fever’ and ‘economic impact of *C. burnetii’*. The Medical Subject Headings (MeSH) keywords related to Q fever were also used to retrieve relevant publications from PubMed. These search terms were tailored to meet the specific language requirements of each database. When Boolean operators were not permitted, we combined the terms as (Q fever) or (*C. burnetii)*. All identified articles that emerged during the searches were exported/uploaded into the Mendeley Reference Manager Software (Mendeley).

### 2.3 Data Screening and Extraction

Mendeley was used to remove duplicate entries on the basis of the author, the year of publication, the title of the article and other publication details. The software was also used to organise references and manage citations for this study. Following the removal of duplicates, the remaining articles were initially screened by title and abstract. All articles deemed eligible for inclusion were retrieved in full text for further assessment. These full‐text articles were assessed for inclusion by two reviewers (L.A. and R.A.). Included articles met the following predetermined inclusion criteria: Research article published between January 2004 and December 2024 that discusses the prevalence (including outbreaks), diagnosis, and/or economic impact of Q fever or *C. burnetii* infections in humans and animals in Africa. Articles on arthropod vectors of *C. burnetii* sampled from animals and humans in Africa were included. Screening was limited to only accessible articles.

Studies conducted outside Africa and/or that were not published in English, including immunology experiments, reviews, editorials, case reports and case‐control studies, were all excluded. Moreover, research focusing on Q fever among returning travellers, diagnostic or therapeutic studies lacking epidemiological data, studies based on theoretical epidemiology, duplicate data published elsewhere and textbooks or manuals were also excluded. Disagreements between the two reviewers were resolved by a third reviewer (N.N.). To ensure consistency and accuracy, a standardised and pre‐piloted form created in Kobo Toolbox was utilised. Retrieved studies were randomly assigned to two groups of reviewers (R.A. and B.T.; E.B. and N.N.). Data from each paper were independently extracted by the members of each group, and any discrepancies were resolved by L.A.

**FIGURE 1:**
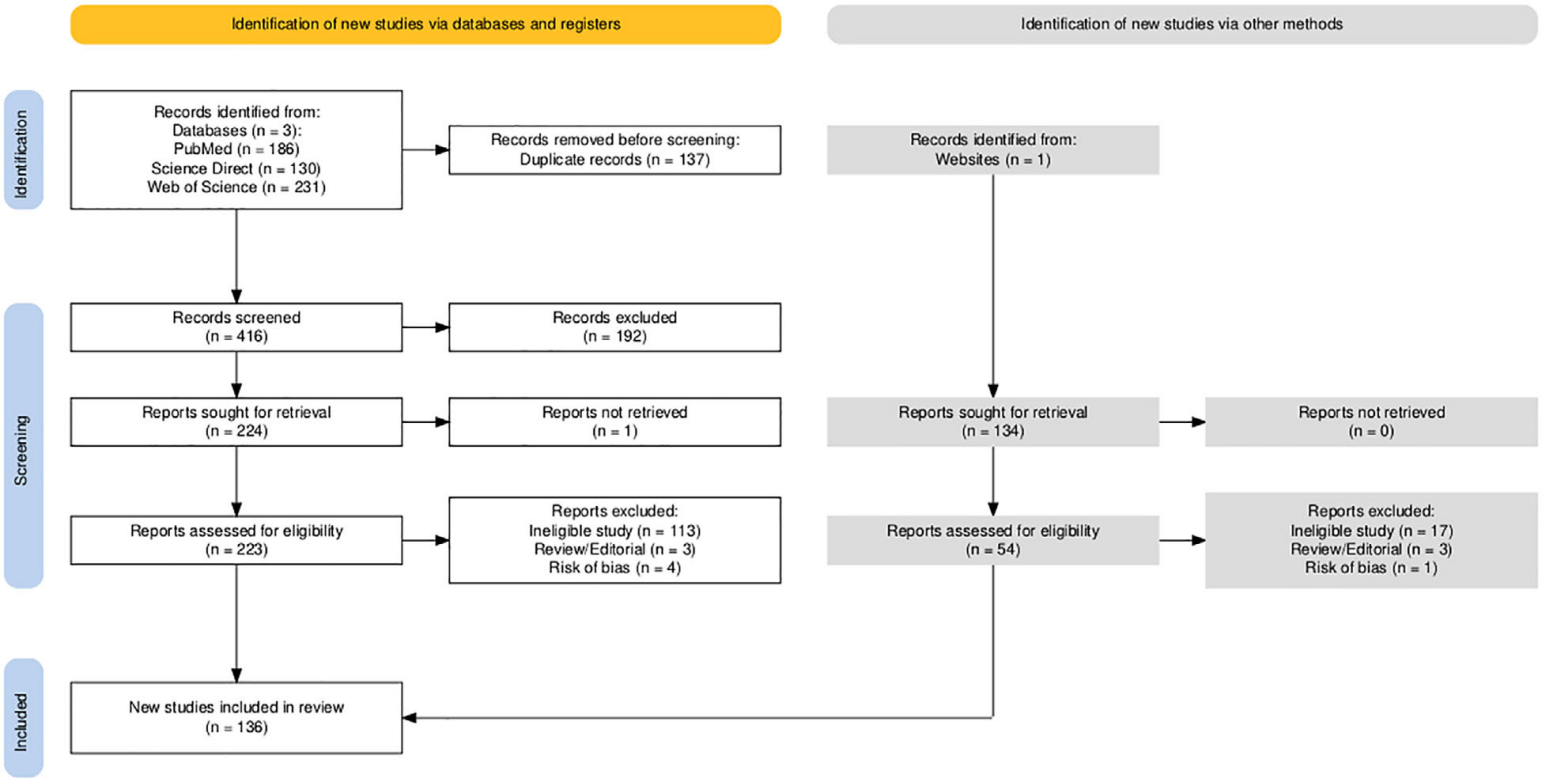
PRISMA 2020 flow diagram for systematic review. Three databases and one website (Google Scholar) were searched to retrieve published papers on Q fever. On the basis of predetermined inclusion and exclusion factors, 136 papers were included in this systematic review. The diagram was generated using the PRISMA 2020 R package and Shiny app [33].

Several studies that were initially considered potentially eligible were excluded during the data extraction phase. A full‐text assessment revealed that these studies were out of scope with respect to predefined inclusion criteria. Some papers were excluded because they did not report primary prevalence data, whereas others lacked extractable data, that is, having a missing numerator or denominator or had a high risk of bias (0–3) (Figure 1), among other reasons. Detailed study characteristics, extracted variables, funding information and country‐level study distribution are provided in Tables S1–S4.

### 2.4 Criteria for Identification and Classification

#### 2.4.1 Prevalence of Q Fever/*C. burnetii*

Studies reporting on the prevalence of Q fever using molecular or serological methods were considered. We included prevalence studies using serological tests that applied specific minimum antibody titre cut‐offs for *C. burnetii* Phase I and/or Phase II antigens as valid evidence of infection [7, 34]. No minimum antibody titre cut‐off was predefined for this review; seropositivity was defined according to the diagnostic thresholds reported in each included study. Therefore, throughout this document, ‘seropositive’ and ‘seropositivity’ refer to serological reactions meeting the defined titre criteria set by each included study.

#### 2.4.2 Economic Burden of Q Fever/*C. burnetii*

We reviewed studies that reported the impacts of Q fever on household finances, human healthcare and livestock production. Our review focused on the direct and indirect economic consequences of coxiellosis in animals, which included factors such as mortality, abortion, mastitis, metritis and decreased productivity. In terms of human healthcare, we considered various aspects, including work absenteeism, costs associated with managing sequelae of Q fever, medical expenses, indirect costs from reduced productivity, costs of preventive measures and financial resources required for implementing surveillance and control measures. Additionally, we looked into the household‐level economic impact and social stigma, such as loss of business for farmers who report abortions or stillbirths.

### 2.5 Risk‐of‐Bias Assessment

The Joanna Briggs Institute (JBI) Critical Appraisal Checklist for Studies Reporting Prevalence Data [35] was used to assess the methodological rigour of each study during data extraction with the Kobo Toolbox. The appropriateness of the sample size, sampling strategy, methods and management of responses of these studies was evaluated. Using this tool, each article was given an overall classification of ‘include’ for scores above 6, ‘exclude’ for scores between 0 and 3 and ‘seek further information’ for scores between 4 and 6. All articles categorised as ‘seek further information’ were reviewed and addressed accordingly.

### 2.6 Data Analysis

Data extracted from the Kobo Toolbox were exported to Excel and organised into the descriptive metadata of the eligible articles, including information on study sites (e.g., country, location, etc.) and populations (e.g., human, livestock, wildlife and arthropods) (Supporting Information 2). Descriptive analyses of the data were conducted using R Studio, version 4.4.3. Data analysis was conducted to summarise prevalence estimates and evaluate heterogeneity across studies. Mean prevalence values, standard deviations (SDs) and coefficients of variation (CVs) were calculated for each host and vector category (Tables S1 and S2). Statistical heterogeneity was assessed using Cochran's *Q* test, *I* ^2^ statistic and Tau‐squared statistic, with *p* values <0.05 indicating statistically significant heterogeneity (Tables S3). *I* ^2^ values greater than 75% were interpreted as indicating substantial heterogeneity among studies. Prevalence estimates were summarised across host groups, including humans, domestic animals, wildlife and arthropod vectors.

The results of the data synthesis were presented in a narrative form, supported by tables and figures, where appropriate, to provide a comprehensive overview of the findings.

## 3 Results

### 3.1 Characteristics of Included Studies

A total of 681 articles were retrieved from the selected databases and Google Scholar for this review. Out of these, 515 papers were excluded on the basis of predetermined inclusion criteria. This left 166 records that were deemed eligible for extraction. After further evaluation, a total of 136 studies met the inclusion criteria (Figure 1). However, one study reported prevalence data for five countries; therefore, country‐specific estimates were extracted separately, resulting in 140 prevalence estimates included in the analysis (Table S1). Nearly all (94.9%) of the reviewed publications were cross‐sectional studies. The characteristics of all included studies and extracted variables are provided in Table S1. Studies were unevenly distributed across the continent, with research concentrated in a limited number of countries, as seen in Figure S1.

Reviewed studies were centred on domestic animals (66.9%), humans (33.1%), arthropods (25.0%) and wildlife (10.3%). With respect to the domestic animals, the studies primarily focused on cattle (42.6%), sheep (33.1%), goats (25.7%), camels (11.0%) and buffaloes (2.2%) (Table S1). The distribution of studies according to host species and population groups is shown in Figure S2. The proportion of studies investigating each host species is also presented in Figure S3.

**FIGURE 2:**
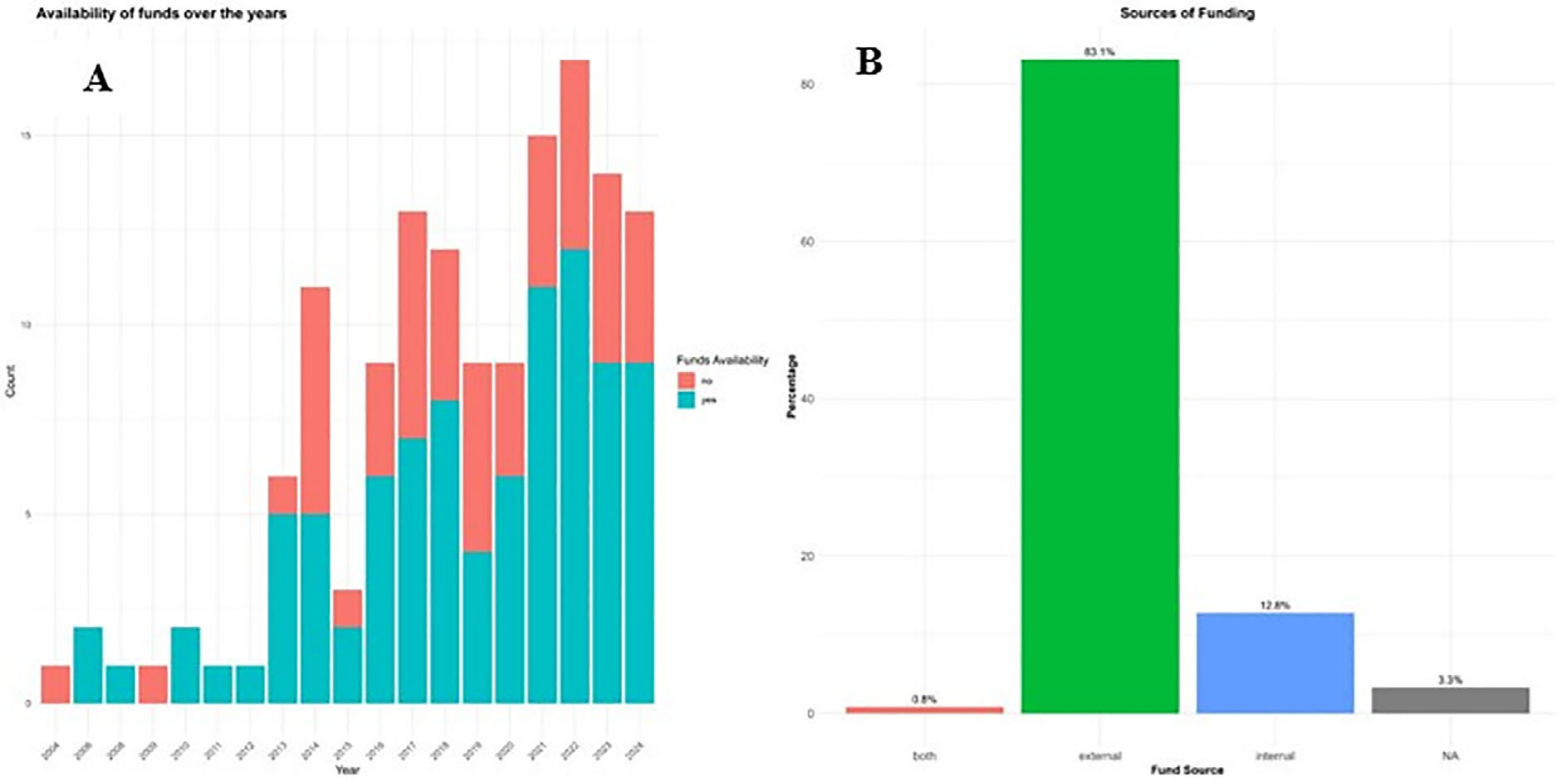
Comparison of funding and sources of funding of Q fever studies reviewed. (A) Temporal trends in funding availability for Q fever research from 2004 to 2024 and (B) distribution of funding sources among reviewed studies. External sources refer to funding from institutions outside of Africa, whereas internal sources refer to funding from within Africa.

Research on Q fever began to increase after 2010, with a significant rise in the number of funded studies following that period. Although many studies reported no financial support, a majority (83.1%) received funding from external sources (institutions outside of Africa) (Figure 2). Only a small percentage of studies (0.8) received funding from both internal sources (within Africa) and external sources, whereas 12.8% relied on internal institutional support, as shown in Figure 2. These findings highlight a heavy reliance on external funding for Q fever research across the continent. Funding characteristics of included studies are summarised in Table S3.

**FIGURE 3:**
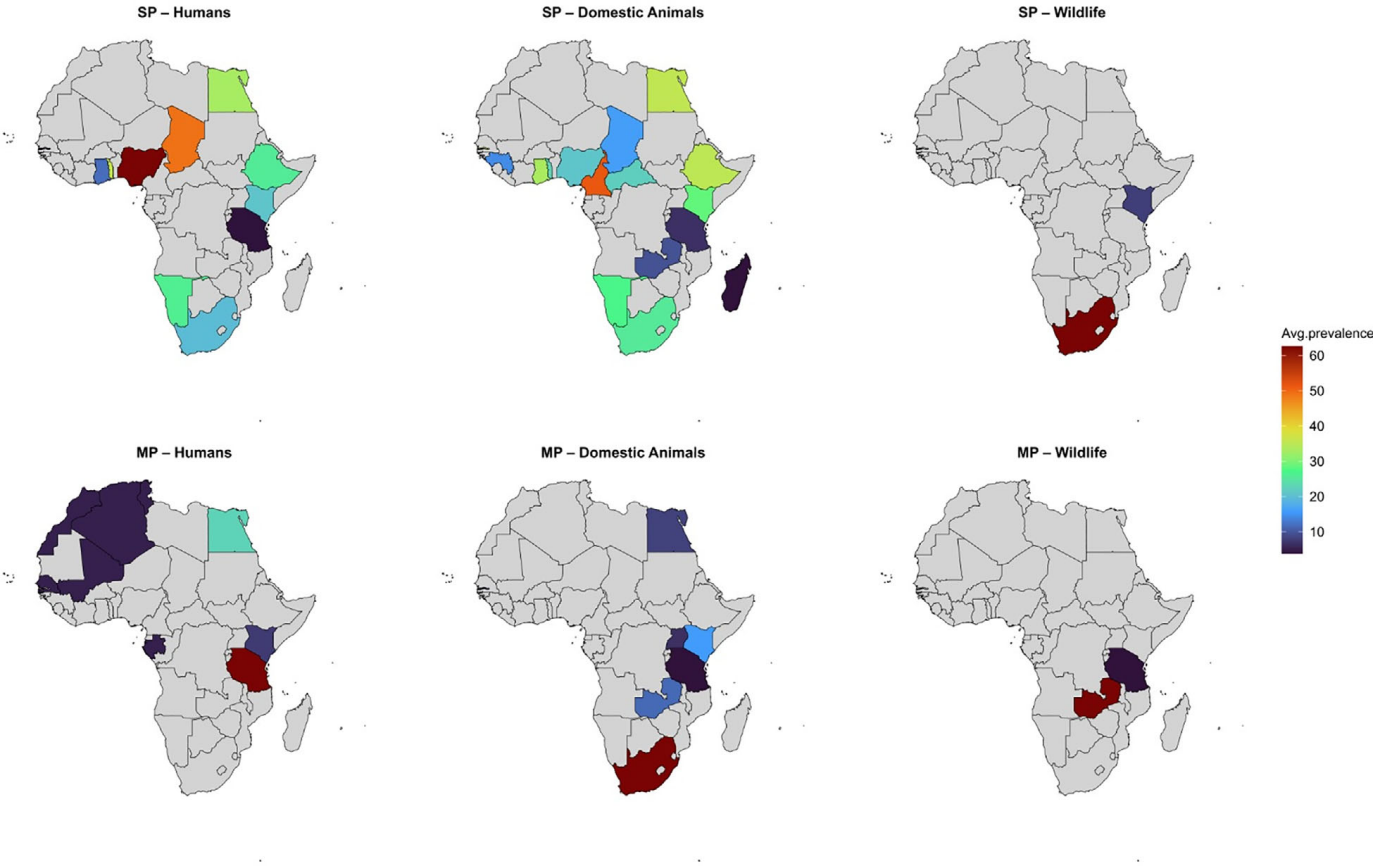
Mean seroprevalence (SP) and molecular prevalence (MP) of Q fever among the various species across Africa. Spatial distribution of average seroprevalence (SP; top row) and average molecular prevalence (MP; bottom row) of *Coxiella burnetii* infection in humans, domestic animals and wildlife across African countries. Warmer colours indicate higher prevalence. Data show substantial geographic variability, with higher SP and MP values observed in selected regions of North, East and Southern Africa, whereas most countries remain unstudied.

Studies included in the analysis employed either molecular or serological methods to assess the prevalence of Q fever, showing significant variation across Africa during the study period (Figure 3). Although it was evident that most African countries (29/54, 53.7%) have not investigated the presence of Q fever, those who did relied primarily on serological approaches, as shown in Table 1. The geographical distribution of studies across African countries is shown in Table S4.

**TABLE 1:** A summary of the number of studies reported per African country and the diagnostic methods used.

Country	sp_human	sp_domestic	mp_human	mp_domestic	mp_arthropods	mp_wild life	sp_wild life	sp_total	mp_total
Algeria	3	8	5	5	6	0	1	12	16
Cameroon	0	2	0	0	1	0	0	2	1
Central African Republic	0	1	0	0	0	0	0	1	0
Chad	1	2	0	0	0	0	0	3	0
Côte d'Ivoire	0	0	0	0	1	0	0	0	1
Egypt	3	5	1	2	3	0	0	8	6
Ethiopia	2	6	0	0	1	0	0	8	1
Gabon	0	0	1	0	0	0	0	0	1
Gambia	1	2	0	1	0	0	0	3	1
Ghana	2	2	0	0	1	0	0	4	1
Guinea	0	1	0	0	0	0	0	1	0
Kenya	5	6	2	2	3	0	1	12	7
Madagascar	0	1	0	0	0	0	0	1	0
Mali	0	0	1	0	3	0	0	0	4
Morocco	0	0	0	0	0	0	1	1	0
Namibia	1	1	0	0	0	0	0	2	0
Nigeria	1	5	0	0	3	0	0	6	3
Sao Tome and Principe	1	0	0	0	1	0	0	1	1
Senegal	0	0	3	0	0	2	0	0	5
South Africa	3	5	0	2	4	0	2	10	6
Tanzania	2	1	3	1	0	2	0	3	6
Togo	1	1	0	0	0	0	0	2	0
Tunisia	0	3	0	2	0	0	0	3	2
Uganda	0	0	0	1	1	0	0	0	2
Zambia	0	1	0	1	0	1	0	1	2
Total	**26**	**53**	**16**	**17**	**28**	**5**	**5**	**84**	**66**

Abbreviations: mp, molecular prevalence; sp, seroprevalence.

**FIGURE 4:**
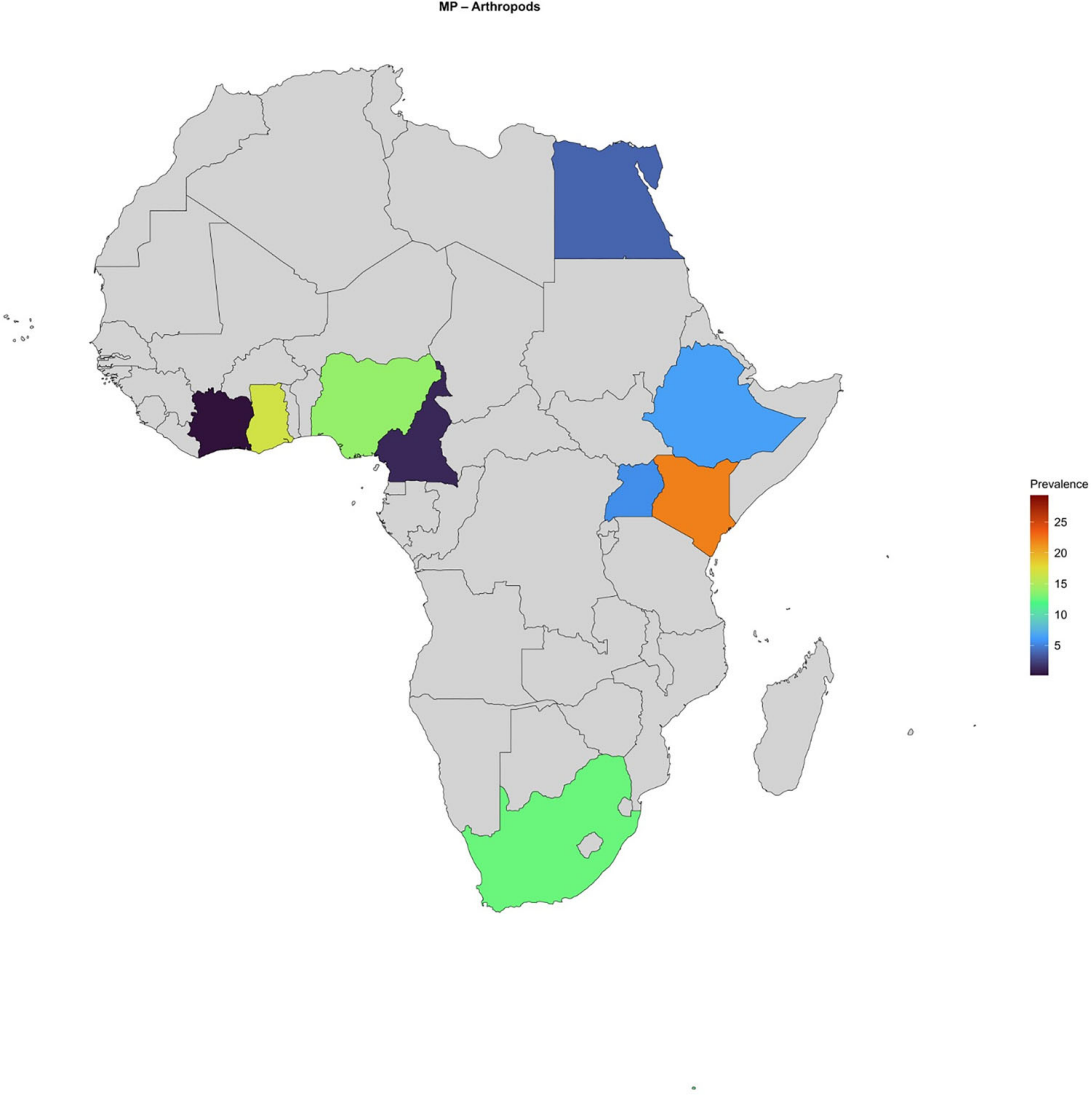
Mean prevalence of Q fever in arthropods. Colours indicate the mean prevalence (%) of infection among sampled arthropods, with warmer colours corresponding to higher prevalence. Grey areas denote countries with no available data.

A number of studies also examined Q fever surveillance in arthropods, as illustrated in Figure 4. Studies on arthropods predominantly focused on ticks (94%). Details of arthropod studies included in the systematic review are presented in Table S2. Tick species examined included *Rhipicephalus*, *Amblyomma* and *Hyalomma* spp. Molecular detection was the primary diagnostic approach used by these studies, most commonly PCR, followed by sequencing (Table S1). All studies reported DNA detection only, with no evidence of active transmission or vector competence.

### 3.2 Prevalence of Q Fever (*C. burnetii*) in Africa

Prevalence estimates varied across host categories and vectors (arthropods) across Africa (Tables S1 and S2). Among domestic animals, mean molecular prevalence ranged from 7.28% (SD: 8.84) in goats to 9.88% (SD: 11.95) in sheep, whereas cattle showed a mean prevalence of 8.27% (SD: 9.73). Humans recorded a mean molecular prevalence of 5.86% (SD: 12.95), whereas pets showed a similar prevalence of 5.77% (SD: 5.17) (Table S3).

**FIGURE 5:**
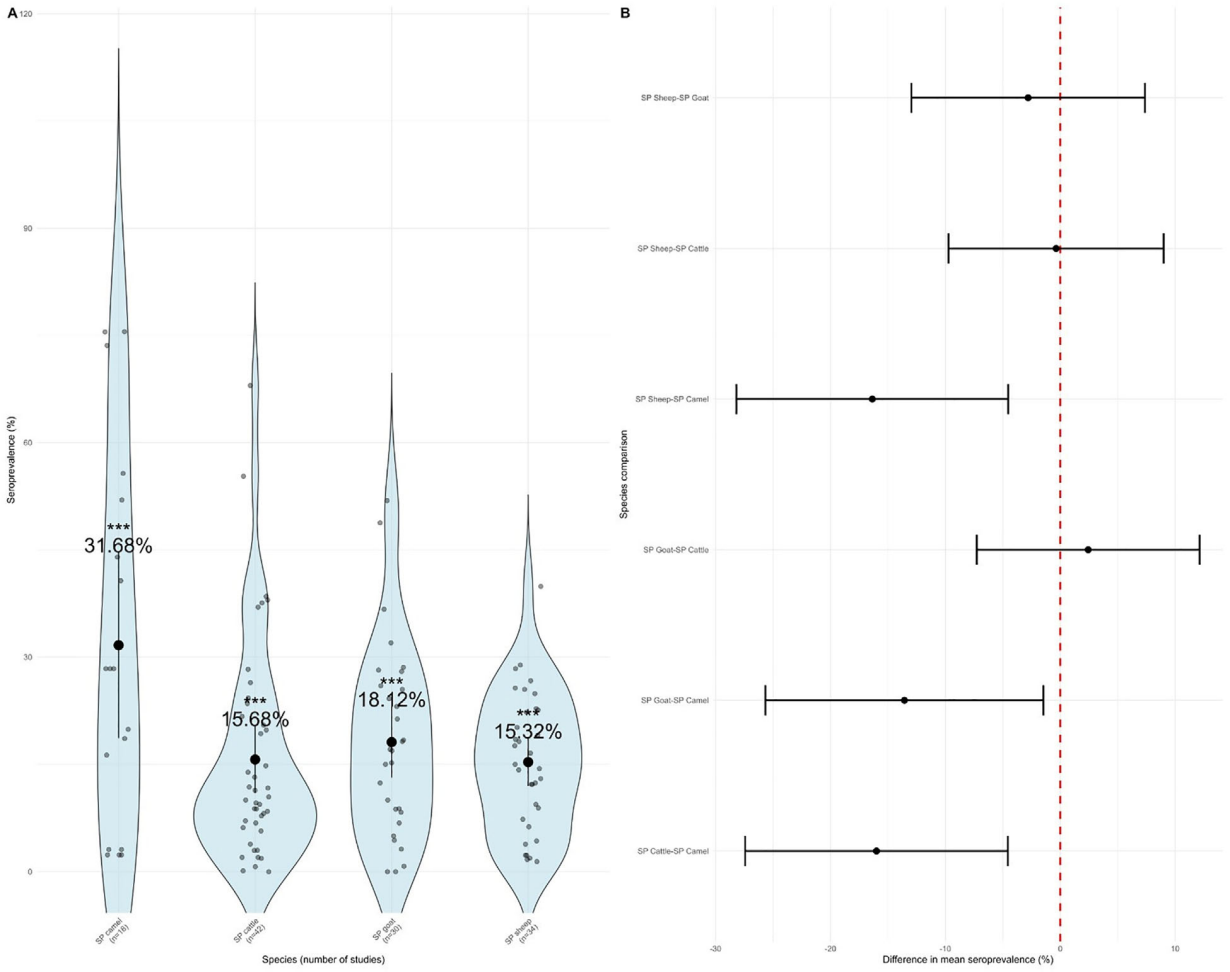
Comparison of mean seroprevalence among livestock. Violin plot showing the distribution of seroprevalence (SP) of *Coxiella burnetii* infection in cattle, goats, sheep and camels across Africa (Table S3). In the left panel (A), the grey dots indicate the individual study‐level seroprevalence estimates, the black dot represents the mean seroprevalence, whereas the vertical line reflects the range of seroprevalence estimates across studies. The asterisks indicate statistically significant differences. The right panel (B) shows pairwise comparisons of species using Tukey's HSD test, showing that camels had significantly higher seroprevalence than other livestock species (***p < 0.001) (see Table S3 for more information). The dashed red line denotes zero difference in mean prevalence. Each line represents the difference in mean seroprevalence between species with 95% confidence intervals.

Generally, seroprevalence estimates were higher across host groups. Camels showed the highest seroprevalence (31.68% ± 26.14%), followed by goats (18.12% ± 13.56%) and humans (21.30% ± 16.39%). Among livestock species, a pairwise comparison of the mean seroprevalence using Tukey's HSD indicated that camels have a significantly higher prevalence than the other livestock species. In summary, the seroprevalence ranking was lowest in sheep, followed by cattle and goats, and highest in camels (see Figure 5).

**FIGURE 6:**
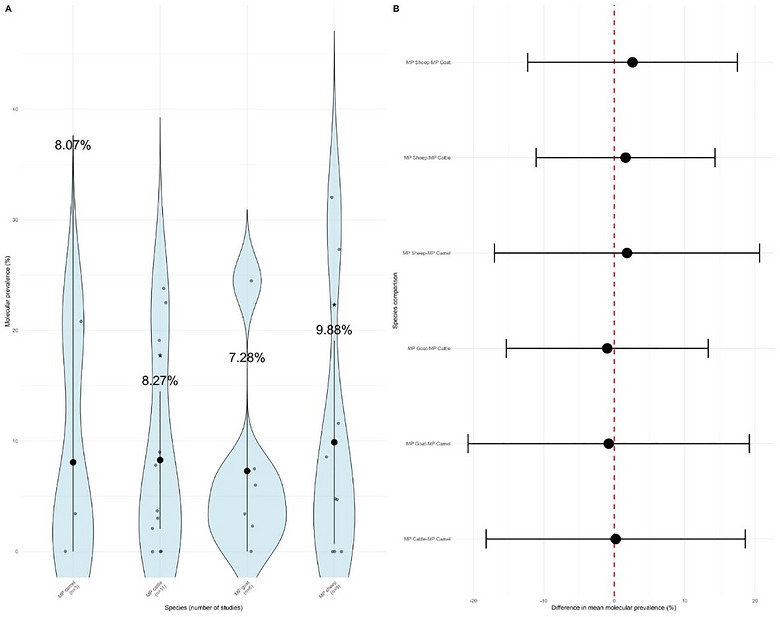
Comparison of mean molecular prevalence among livestock. Violin plot showing the distribution of molecular prevalence (MP) of *Coxiella burnetii* infection in cattle, goats, sheep and camels across Africa (Table S3). In the left panel (A), the grey dots indicate the individual study‐level molecular prevalence estimates, the black dot represents the mean molecular prevalence, whereas the vertical line reflects the range of molecular prevalence estimates across studies. Pairwise comparisons of species (right panel, B) were conducted using Tukey's HSD test. Each line represents the difference in mean molecular prevalence between species with 95% confidence intervals. The dashed red line denotes zero difference in mean prevalence. Increased variability in camels complicates the determination of significant differences between the species.

For molecular prevalence, samples tested included blood, milk and vaginal swabs. Although variability in the molecular prevalence among camels made it challenging to detect statistically significant differences when comparing them to other species, sheep exhibited the highest average prevalence (9.88% ± 11.95%). As a result, statistical differences are only noticeable between sheep (9.88%) and goats (7.28%) (Figure 6).

Wildlife populations recorded a mean molecular prevalence of 18.92% (SD: 18.85), suggesting a possible role as reservoirs of infection. Vector‐related prevalence was also significant, particularly among ticks with mean molecular prevalence estimates of 11.70% (SD: 16.24) (Table S3).

Substantial between‐study heterogeneity was observed across all host groups and species. Using the *I* ^2^ statistic and Cochran's *Q* test, most categories showed very high heterogeneity (*I* ^2^ > 90%), including arthropods (97.2%), domestic animals (98.8%), humans (97.9%) and wildlife (92.9%) (*p* < 0.001) (Table S3). Similarly high heterogeneity was observed in both molecular and serological estimates across species. For example, serological studies in cattle (97.6%), goats (96.6%), sheep (97.0%) and humans (96.2%) showed substantial variability between studies. Molecular estimates also demonstrated high heterogeneity in arthropods (96.4%), ticks (94.7%), domesticated animals (94.1%) and humans (93.8%). Overall, the consistently high heterogeneity likely reflects differences in host species, ecological settings, diagnostic approaches and study design across the included studies. Consequently, random‐effects models were considered appropriate for the quantitative synthesis (Table S3).

### 3.3 Evidence of Zoonotic Potential of *C. burnetii* Across Africa

Several studies (94%) examined the zoonotic potential of *C. burnetii* at the human–domestic animal interface. Evidence was primarily based on parallel or separate detection of *C. burnetii* in humans and domestic or wild mammals within the same geographic settings. Human studies commonly focused on occupationally exposed populations, whereas animal studies reported serological or molecular detection in livestock and wildlife species. Cross‐species interaction was inferred from shared environments, livestock ownership, animal husbandry practices or occupational exposure rather than from direct measurement of transmission events.

### 3.4 Economic Impact and Potential Economic Burden of Q Fever in Africa

Of the 136 articles reviewed, none assessed the economic burden or impact of Q fever on livestock production, public health or veterinary health, even though they all established the presence of *C. burnetii* in different hosts (animals, humans and arthropods). Most of these articles made limited references to the potential economic impact or implications of the disease, but none attempted a cost‐benefit analysis.

### 3.5 Risk of Bias of Included Studies

Methodological quality was assessed for all the included studies. Overall, the studies were judged to have a low risk of bias using the JBI Critical Appraisal Checklist for Studies Reporting Prevalence Data. Common strengths of these studies included an appropriate sample frame, adequate sample size and use of valid methods for the identification of the condition. Frequent limitations were about data coverage sufficiency and insufficient reporting of response rate (Table S1).

## 4 Discussion

This systematic review highlights the widespread presence of Q fever across several African nations. Despite this predominance, Q fever remains largely overlooked, underscoring the need for increased attention to this debilitating disease. Although Q fever has been reported in multiple African countries, research efforts were mainly concentrated in Algeria, South Africa, Egypt, Kenya, Ethiopia and Tanzania. As a neglected tropical disease, Q fever appears to receive considerably less funding than other priority diseases such as malaria, HIV/AIDS and tuberculosis. This observation is supported by Mwoka [36], who further notes a shift in funding priorities towards areas such as pandemic preparedness and response, general diagnostics and advancements in vaccine delivery technologies.

The reviewed articles included in this systematic review reported varying prevalence of Q fever in livestock, including sheep, goats, cattle, camels and arthropods. However, cattle, sheep and goats were the most studied species, with most research conducted in areas where these animals were mainly reared. This probably reflects the economic importance of these animals to the livestock industry and the impact of coxiellosis on their productivity [37, 38, 39].

Regarding arthropods, ticks were reported as important vectors of *Coxiella* in Africa in included studies [21, 40]. Several papers reported herds of animals infested with ticks that tested positive for *Coxiella* [41, 42, 43]. Mediannikov et al. [44] found *C. burnetii* DNA in 37.6% of tick specimens, whereas Diarra et al. [45] identified the bacteria in six tick species in Mali, with an overall prevalence of 33.4%. Although Honarmand [18] noted that the transmission of *C. burnetii* to humans remains poorly understood, a single study in Algeria found human body lice to be positive for *C. burnetii* [46], suggesting the necessity for additional research on their potential role in transmitting Q fever in Africa. Ticks may play a role in the ecology of *C. burnetii*, although evidence from prevalence studies is largely based on molecular detection and does not confirm vector competence. Moreover, several studies reporting *Coxiella* in ticks indicate a significant challenge for the livestock industry and underscore the need to investigate their role in transmitting this parasite to humans.

The potential for reported *C. burnetii* positives in ticks to actually represent *Coxiella*‐like endosymbionts (CLEs) is also a significant concern in epidemiological research [47, 48]. These non‐pathogenic bacteria are widespread among tick genera and share genetic similarities with *C. burnetii*, complicating accurate identification [47, 49]. For example, the study by Hsi et al. [48] in Sao Tome found *C. burnetii* undetected in ticks, yet CLEs were present in 99% of specimens, highlighting the risk of overestimating the pathogen's prevalence. Similarly, CLEs were identified in northern Kenya by Getange et al. [12]. Although several studies employed molecular assays targeting IS1111, this insertion sequence is not species‐specific and has been reported in diverse CLEs associated with ticks [47]. Consequently, detection based solely on IS1111 may result in misclassification and overestimation of *C. burnetii* occurrence. Reliable identification, therefore, requires the use of additional confirmatory targets and/or sequencing‐based approaches [50, 51], as was observed in 68% of the studies on ticks (Table S2). The inability of some studies to distinguish *C*. *burnetii* from CLEs represents an important limitation and may have contributed to inflated prevalence estimates reported in tick populations.

For the most part, studies included in this review used serological methods, which align with findings from Diseko et al. [52], who also observed that serological assays were commonly used in Q fever studies. Biological samples commonly used for diagnosis in the included studies comprised blood, arthropods and spleen tissue. Blood was the most frequently sampled material, enabling serological detection of antibodies against *C. burnetii*. This is consistent with Bwatota et al.’s [53] finding, who noted that serological methods are often preferred due to the limited availability of molecular diagnostics in Africa. Furthermore, serological tests are cost‐effective and suitable for field conditions in Africa, making them ideal for large‐scale epidemiological studies. However, interpretation of these seroprevalence data is subject to several limitations. Serological assays primarily indicate prior exposure to C. burnetii and do not distinguish between past and active infection, potentially leading to overestimation of current infection prevalence. Variation in serological assays and antibody titre cut‐offs used to define *C. burnetii* seropositivity across studies likely contributed to differences in reported prevalence estimates. Even though all the studies used established serological assays, including commercial ELISA kits, a few (9/74, 12.2%) did not provide adequate assay validation parameters or manufacturer performance characteristics, which may impact the study findings.

The public health impacts of Q fever are substantial, particularly in developing nations. This disease poses a zoonotic risk to individuals working in the farming sector, including livestock farmers, animal handlers, animal researchers and veterinarians [54]. Spontaneous shedding of *C. burnetii* in human breast milk and faecal samples was also reported by Mediannikov et al. [44]. The findings from Mediannikov et al. [44] indicate that humans may act as potential reservoirs for *C. burnetii*, similar to other mammals. This is supported by Noden et al. [55], who documented a *C. burnetii* seroprevalence of 26.1% among volunteer blood donors. Prior research conducted in Nigeria also indicated 44% of blood donors were positive for *C. burnetii* antibodies [56].

In addition, studies conducted in Tanzania, Tunisia, Burkina Faso and other African nations indicated that *C. burnetii* infections may contribute significantly to hospital admissions for febrile illnesses [57, 58, 59]. The articles on humans suggested that there have been instances where clinicians did not suspect this disease in patients who later tested positive. This issue is compounded by the frequent misdiagnosis of Q fever as typhoid fever, malaria, pneumonia or undifferentiated fever [60, 61, 62]. Recognising this is crucial for effective disease outbreak management. If left untreated, Q fever can lead to severe health complications, developing into chronic Q fever and resulting in more serious complications such as endocarditis, organ damage, infertility and neurological effects [50, 63]. The cost associated with managing Q fever infections, along with the aftermath of the disease, could lead to increased medical expenses, work absenteeism and additional burden on healthcare resources.

Q fever significantly affects livestock, posing economic challenges, especially in rural African communities where farming is a key income source and social status indicator. This concern is heightened by the global importance of livestock production, which employs around 1.3 billion people worldwide [25, 64]. Livestock farming in Africa also contributes to sustainable development goals, supporting zero hunger (SDG 2), poverty reduction (SDG 1) and decent work and economic growth (SDG 8) by providing food, income and employment for millions. Q fever causes issues like abortions, which can occur at any stage, and stillbirths, which lead to significant economic losses [9, 65]. These reproductive challenges contribute to the socioeconomic burden of Q fever infections [66]. Nevertheless, the economic burden of zoonotic diseases remains largely unavailable, and this was evident in the articles reviewed. Although several studies in Africa have reported on the impact of Q fever on the livelihoods of farmers, infected individuals and public health, none have quantitatively assessed important economic impact indicators such as direct and indirect costs, estimates of the disease burden, economic modelling and risk mitigation costs. Further investigations into the economic impact of Q fever on both human and animal health are necessary to assess the costs associated with this disease.

Finally, we observed critical knowledge and methodological gaps that impede the complete appreciation of the Q fever dynamics in Africa. The strong concentration of studies in a few countries, which rely on serological detection methods, is suggestive of geographical and diagnostic biases that might mask the actual burden of the disease on the continent. That is, active infection is grossly underestimated, obscuring transmission pathways. The co‐occurrence of *C. burnetii* in humans and animal hosts within shared settings suggests zoonotic potential; however, most studies did not directly measure transmission pathways, highlighting a key evidence gap. The absence of research at the human–domestic–wildlife interface shows a major gap in the acceptance and implementation of the One Health approach. Addressing these gaps will require harmonised One Health surveillance systems and expanded diagnostic capacity across Africa.

## 5 Limitation

A key limitation of this review is the heterogeneity and scarcity of available data across countries. Epidemiological studies were lacking in many African countries, with a few concentrated in a few regions, creating geographical gaps that restricted continent‐wide generalisability. Additionally, many included studies relied on serological methods; the variability in serological assays, diagnostic cut‐offs and test performance across studies and host species further limits comparability of prevalence estimates.

Failure of included studies to differentiate *Coxiella* from CLEs may result in overestimation of *C. burnetii* detection in ticks. As CLEs are common in ticks, studies relying solely on non‐specific PCR targets without confirmatory assays may overestimate the presence of *C. burnetii*. This limits the interpretation of tick infection data and underscores the need for improved molecular standardisation.

Furthermore, few studies (3/136) integrated human, animal and vector data concurrently, limiting insights into the full One Health dynamics of transmission. Lastly, none of the studies quantified the economic impact of Q fever. The presence of methodological limitations identified through the JBI appraisal, particularly related to data coverage sufficiency and insufficient reporting of response rate, should be considered when interpreting the reported prevalence estimates. Studies lacking extractable data, such as missing numerator or denominator, were excluded because prevalence could not be calculated. Although methodologically necessary, it may have introduced selection bias and led to the omission of relevant findings. The high heterogeneity values observed across host groups, species and diagnostic approaches (*I* ^2^ > 90%, *p* < 0.001) underscore the substantial variability in prevalence estimates and highlight the need for cautious interpretation of the pooled results.

Together, these limitations highlight the need for more comprehensive and standardised surveillance efforts to improve understanding of the epidemiology and economic impact of Q fever in Africa.

## 6 Conclusion

This systematic review demonstrates the widespread prevalence of Q fever infection throughout Africa, highlighting its significant threat to both human and animal health. It also reveals a complex dynamic, where Q fever is sustained by diverse reservoirs and potential reservoir hosts, with livestock serving as the dominant source of human infection. The presence of *C. burnetii* in arthropods, confirmed by molecular analysis in several tick species, also illustrates the pathogen's ecological resilience. Most studies demonstrate that exposure risks extend beyond direct animal contact, encompassing environmental contamination and vector‐borne transmission. These observations underscore the necessity of a One Health approach.

Considering the serious implications of *C. burnetii* for human and animal health, as well as its economic consequences, research is needed to investigate the economic impacts of *C. burnetii* and to establish robust disease surveillance programmes in both animal and human populations. These surveillance efforts are critical for designing effective control strategies. Further investigation is also needed to explore the role of Q fever in febrile illnesses, especially in areas with significant populations of livestock and companion animals. Ultimately, research findings should inform public health measures aimed at reducing transmission risks in both animals and humans, thereby lessening the disease's overall impact on the continent. The presence of infection in livestock, humans, wildlife and arthropods underscores the complex epidemiology of *C. burnetii* and reinforces the importance of a One Health approach for surveillance and control.

## Author Contributions

**Linda Ama Owusuaa Amoah**: conceptualisation, methodology, investigation, data curation, writing – original draft, writing – review and editing, project administration. **Richard Kwamena Abbiw**: conceptualisation, methodology, investigation, data curation, data analysis, writing – original draft, writing – review and editing. **Naa Awula Narkie Nartey**: conceptualisation, methodology, investigation, data curation, writing – original draft, writing – review and editing. **Becky Ewurama Tetteh**: investigation, data curation, writing – original draft, writing – review and editing. **Elizabeth Bugase**: investigation, data curation, writing – original draft, writing – review and editing.

## Funding

The authors have nothing to report.

## Ethics Statement

The authors have nothing to report.

## Conflicts of Interest

The authors declare no conflicts of interest.

## Supporting information



Supporting‐information‐puh270323‐sup‐0001‐SuppMat.docx

Supporting‐information‐puh270323‐sup‐0002‐TableS4.xlsx

Supporting‐information‐puh270323‐sup‐0003‐Data.pdf

Supporting‐information‐puh270323‐sup‐0004‐Prisma.docx
**FIGURE S1 Geographic distribution of Q fever studies conducted across Africa (2004–2024)**. Distribution of studies included in the systematic review by African country. The figure illustrates the geographical coverage of Q fever research across the continent and highlights disparities in research output among countries.
**FIGURE S2 Number of Q fever studies by host species and population group**. Number of studies investigating Q fever in humans, domestic animals, wildlife and arthropods. The figure summarises the relative research attention given to different host groups included in the review.
**FIGURE S3 Proportion of studies investigating specific host species and populations**. Percentage of included studies reporting Q fever in specific host species or population groups. Red bars indicate estimates derived from more than two studies, whereas blue bars represent estimates based on two or fewer studies.
**TABLE S1. Molecular prevalence of Q fever reported across African countries**.
Country‐specific molecular prevalence estimates of *Coxiella burnetii* in humans, domestic animals, wildlife, arthropods and selected animal species. Values are presented as mean prevalence (%) ± standard deviation (SD) where multiple studies were available. Domestic represents the aggregate prevalence across domestic animal species included in the analysis. Variability could not be calculated when only one study was available (*n* = 1).
**Table S2. Seroprevalence of Q fever reported across African countries**.
Country‐specific seroprevalence estimates of *Coxiella burnetii* in humans, domestic animals, wildlife and selected animal species. Values are presented as mean prevalence (%) ± standard deviation (SD) where multiple studies were available. Domestic represents the aggregate prevalence across domestic animal species included in the analysis.
**Table S3 Assessing statistical heterogeneity among studies using Cochran's *Q* test, *I* ^2^ statistics and** *τ* ^2^.
Measures of heterogeneity for molecular and serological prevalence estimates across host groups and species. Heterogeneity was assessed using Cochran's *Q* statistic, *Q p* value, *I* ^2^ statistic and *τ* ^2^. Mean prevalence, standard deviation (SD) and coefficient of variation (CV) are also presented. Domestic represents aggregate prevalence across domestic animal species, whereas arthropod represents aggregate prevalence across arthropod vectors included in the analysis.
**TABLE S1 Characteristics of studies included in the systematic review of Q fever in Africa (2004–2024)**.
This table contains the complete dataset extracted from the 140 prevalence estimates included in the review. Information includes study characteristics (authors, year, country and study design), host populations, sample types, diagnostic methods, prevalence estimates (serological and molecular), reported risk factors and risk‐of‐bias assessments based on the Joanna Briggs Institute (JBI) checklist.
**TABLE S2 Characteristics of arthropod‐based studies investigating *Coxiella burnetii* in Africa**.
This table summarises studies reporting molecular detection of *Coxiella burnetii* in arthropods, including ticks, fleas and other vectors. Variables include study location, arthropod species, sample size, diagnostic methods, prevalence estimates, reported risk factors and risk‐of‐bias assessments.
**TABLE S3 Funding sources and funding characteristics of studies included in the review**.
This table summarises funding information reported by included studies, including funding source, funding type (internal, external or both) and geographic origin of funding, funding agency, and grant, contract, work‐unit or award numbers where available.
**TABLE S4 Distribution of Q fever studies across African countries by host group and diagnostic approach**.
This table presents the number of studies conducted in each African country, stratified by host group (humans, domestic animals, wildlife and arthropods) and diagnostic approach (serological and molecular). Totals are provided to illustrate the geographical distribution of Q fever research across the continent.

## Data Availability

The corresponding author will provide the data supporting this study's conclusions upon request.
