# Supplementary material for: Q Fever in Africa: A Systematic Review of Prevalence, Zoonotic Potential and Disease Burden
Source: Public Health Chall. 2026 Aug 5;5(3):e70323. doi: 10.1002/puh2.70323 (PMC13439519; doi:10.1002/puh2.70323)
Supplement: Supplementary file 1 [file PUH2-5-e70323-s001.pdf]

Clone of Q fever: a systematic review of prevalence and burden in Africa 1.2

Select the extractor

Who is filling this form

- Linda
- Naa
- Kwamena
- Becky
- Liz

Information about the Paper

1. Study Title

Please copy title and paste

---

2. Authors

Please copy and paste all the authors

---

3. Year of publication

2004 to 2024

---

4. Journal

Journal name

---

5. DOI

copy and paste

---

18. Source of funding

- Yes
- No

Source of funding

state the funding source

Information on the study site(s)

Country/countries

select all the countries where the study was carried out

- [ ] Algeria
- [ ] Angola
- [ ] Benin
- [ ] Botswana
- [ ] Burkina Faso
- [ ] Burundi
- [ ] Cabo Verde/ Cape Verde
- [ ] Cameroon
- [ ] Central African Republic
- [ ] Chad
- [ ] Comoros
- [ ] Democratic Republic of the Congo
- [ ] Republic of Congo
- [ ] Djibouti
- [ ] Egypt
- [ ] Equatorial Guinea
- [ ] Eritrea
- [ ] Eswatini/Swaziland
- [ ] Ethiopia
- [ ] Gabon
- [ ] Gambia
- [ ] Ghana
- [ ] Guinea
- [ ] Guinea Bissau
- [ ] Ivory Coast/ Cote d'Ivoire
- [ ] Kenya
- [ ] Lesotho
- [ ] Liberia
- [ ] Libya
- [ ] Madagascar
- [ ] Malawi
- [ ] Mali
- [ ] Mauritius

- [ ] Mauritania
- [ ] Morocco
- [ ] Mozambique
- [ ] Namibia
- [ ] Niger
- [ ] Nigeria
- [ ] Rwanda
- [ ] Sao Tome and Principle
- [ ] Senegal
- [ ] Seychelles
- [ ] Sierra Leone
- [ ] Somalia
- [ ] South Africa
- [ ] South Sudan
- [ ] Sudan
- [ ] Tanzania
- [ ] Togo
- [ ] Tunisia
- [ ] Uganda
- [ ] Zambia
- [ ] Zimbabwe

Setting

where was the study conducted e.g, farm, hospital etc.

- [ ] Hospital
- [ ] Communities
- [ ] In the wild
- [ ] Farm
- [ ] Other setting

description of the area sampled

- [ ] urban
- [ ] rural

If other settings,

---

8. Economic factors

Summarize the burden if any

Populations studied

Description of population

- [ ] Human
- [ ] Domestic animals
- [ ] Wildlife
- [ ] Arthropods and Insects

Specific domestic animals

- [ ] Sheep
- [ ] Goat
- [ ] Cattle
- [ ] Dog
- [ ] Poultry
- [ ] Camel
- [ ] Buffalo
- [ ] others

other domestic animals

Specific wildlife

Specific arthropods and insects

- [ ] Ticks
- [ ] Fleas
- [ ] Lice
- [ ] Other arthropods

other arthropods

Sources of arthropods and insects

from where did they pick the arthropods

- [ ] livestock
- [ ] poultry
- [ ] dogs
- [ ] cats
- [ ] wildlife
- [ ] camels/dromedary
- [ ] donkey
- [ ] Humans
- [ ] others

Demographics of human population

Human sample size

males

females

Human age ranges

Ethnicity

values

☐ children (<18yr) ☐ adult (18 -60yr) ☐ senile (>60yr)

Demographics of livestock population (combine all of them)

Total livestock

females livestock

male livestock

age range of livestock

livestock values

☐ young (<1yr) ☐ adult (1 -5yr) ☐ aged (>5yr)

Sample sizes of wildlife and arthropods

wildlife

arthropods

Sample size

Methods used

Duration of study (yrs)

when was the study conducted

Study design

- ○ Cross sectional
- ○ Case series
- ○ retrospective
- ○ Others

other study designs

Nature of samples

- ☐ blood
- ☐ vaginal swab
- ☐ arthropod
- ☐ faeces
- ☐ milk
- ☐ other tissues

if other tissues,

Diagnostic tool

- ☐ ELISA
- ☐ PCR e.g., qpcr, pcr, rtpcr etc
- ☐ Culture dependent sequencing
- ☐ Culture
- ☐ culture independent sequencing (metagenomics)
- ☐ other diagnosis
- ☐ Sequencing post PCR

other diagnosis

Inclusion criteria

Exclusion criteria

Results: prevalence, risk and burden

Was the prevalence obtained from multiple countries

- yes
- no

State the country specific prevalence

e.g., Ghana = 12.5, Togo = 5.6,

overall prevalence

overall (humans)

overall (livestock)

overall (wildlife)

overall (arthropods)

seroprevalence

molecular prevalence

p value

animal specific prevalence (just interested in the livestock, poultry and pets

sheep

cattle

goat

camel

Buffalo

dog and cat

poultry

Equine

seroprevalence

molecular prevalence

prevalence of athropods

ticks

fleas

others

prev_athro

Identified risk

- [ ] Age (animal)
- [ ] sex (animal)
- [ ] Age (human)
- [ ] Sex (human)
- [ ] Species of animal
- [ ] Breed
- [ ] Proximity of animals to human settlement
- [ ] Season
- [ ] Disease status in humans
- [ ] other risks

other risks

---

what animal sex is the most susceptible/ at risk

- ( ) male animals
- ( ) female animals

what human sex is the most susceptible/at risk

- ( ) male humans
- ( ) female humans

what human age is the most susceptible/at risk

- ( ) children
- ( ) adult
- ( ) aged

what animal age is the most susceptible/at risk

- ( ) young animals
- ( ) adult animals
- ( ) senile animals

what animal species are considered the most susceptible/ at risk
choose the top 3 susceptible animals in the decreasing order of susceptibility/risk

---

1st choice

- ( ) Sheep
- ( ) Goat
- ( ) Cattle
- ( ) Camel
- ( ) Dogs
- ( ) Cats
- ( ) Poultry
- ( ) Buffalo
- ( ) Other

2nd choice

- Sheep
- Goat
- Cattle
- Camel
- Dogs
- Cats
- Poultry
- Buffalo
- Other

3rd choice

- Sheep
- Goat
- Cattle
- Camel
- Dogs
- Cats
- Poultry
- Buffalo
- Other

what is the minimum distance to an infected animal/human to be at risk (give answer in metre)...just the number. // 1km = 1000m

when (season) is the risk highest

- dry
- wet/rainy
- others

which disease status poses the most risk to humans

- acute infectious diseases
- chronic infectious diseases
- non-infectious diseases e.g., diabetes etc.
- others

Study limitations

What is the burden of Q fever

summarize the identified burden as proposed by the authors (from results or discussion attributable to the authors)

Risk of Bias

**Risk of bias determinant** *fill this per paper*	Yes	No	Unclear	Not applicable
**Was the sample frame appropriate to address the target population?**	○	○	○	○
**Were study participants sampled in an appropriate way?**	○	○	○	○
**Was the sample size adequate?**	○	○	○	○

**Were the study subjects and the setting described in detail?**	◯	◯	◯	◯
**Was the data analysis conducted with sufficient coverage of the identified sample?**	◯	◯	◯	◯
**Were valid methods used for the identification of the condition?**	◯	◯	◯	◯
**Was the condition measured in a standard, reliable way for all participants?**	◯	◯	◯	◯
**Was there appropriate statistical analysis?**	◯	◯	◯	◯
**Was the response rate adequate, and if not, was the low response rate managed appropriately?**	◯	◯	◯	◯

Overall appraisal

- ◯ Include (>5 yes)
- ◯ Exclude (<3 yes)
- ◯ Seek further Information (3 - 5 yes)

Reviewer's comments

Reviewer's comments

Further reading

suggest any paper you wish we read based on this paper. preferably, use the DOI or first author, year and title

---
