# Supplementary material for: Q Fever in Africa: A Systematic Review of Prevalence, Zoonotic Potential and Disease Burden
Source: Public Health Chall. 2026 Aug 5;5(3):e70323. doi: 10.1002/puh2.70323 (PMC13439519; doi:10.1002/puh2.70323)
Supplement: Supplementary file 2 [file PUH2-5-e70323-s003.docx]

**SUPPORTING INFORMATION**

**Figures**

**Figure S 1. Geographic distribution of Q fever studies conducted across Africa (2004-2024**)

Distribution of studies included in the systematic review by African country. The figure illustrates the geographical coverage of Q fever research across the continent and highlights disparities in research output among countries.

**Figure S 2. Number of Q fever studies by host species and population group**

Number of studies investigating Q fever in humans, domestic animals, wildlife, and arthropods. The figure summarizes the relative research per different host groups included in the review.

The red indicates data derived from more than two studies, while blue represents estimates based on two or fewer studies.

**Figure S 3. Percentage of studies investigating specific species or populations**

Percentage of included studies reporting Q fever in specific host species or population groups. Red bars indicate estimates derived from more than two studies, whereas blue bars represent estimates based on two or fewer studies.

**Tables**

**Table S 1. Molecular prevalence of Q fever recorded by studies across Africa**

| Country | Humans  Mean ± SD | Domestic  Mean ± SD | Wildlife  Mean ± SD | Arthropods  Mean ± SD | Ticks  Mean ± SD | Sheep  Mean ± SD | Cattle  Mean ± SD | Goat  Mean ± SD | Equine  Mean ± SD | Camel  Mean ± SD | Pets  Mean ± SD |
| --- | --- | --- | --- | --- | --- | --- | --- | --- | --- | --- | --- |
| Algeria | 6.09 ± 7.10 | 9.93 ± 9.73 | 52.43 | 4.23 ± 5.31 | 7.31 ± 7.03 | 4.29 ± 6.06 | 38.53 ± 51.10 | 0.00 | - | - | 52.50 ± 74.25 |
| Cameroon | - | - | - | 1.20 | - | - | - | - | - | - | - |
| Central African Republic | - | - | - |  | - | - | - | - | - | - | - |
| Chad | - | - | - |  | - | - | - | - | - | - | - |
| Côte d'Ivoire | - | - | - | 0.20 | - | - | - | - | - | - | - |
| Egypt | 5.60 | 4.75 ± 5.44 | - | 3.79 ± 3.93 | 4.22 ± 5.46 | 0.00 | 1.85 ± 2.62 | 3.40 | 1.60 | 0.00 | 7.30 |
| Ethiopia | - | - | - | 6.40 | 6.40 | - | - | - | - | - | - |
| Gabon | 0.00 | - | - |  | - | - | - | - | - | - | - |
| Gambia | - | 3.00 | - |  | - | - | - | - | - | - | - |
| Ghana | - | - | - | 16.70 | 11.23 ± 7.73 | - | - | - | - | - | - |
| Guinea | - | - | - |  | - | - | - | - | - |  | - |
| Kenya | 1.10 ± 1.56 | 11.34 ± 13.39 | - | 22.08 ± 21.00 | 2.50 | 2.35 ± 3.32 | 2.10 | 2.30 | - | 12.10 ± 12.30 | 2011.12 |
| Madagascar | - | - | - |  | 1.00 ± 0.00 | - | - | - | - | - | - |
| Mali | 5.10 | - | - | 29.32 ± 25.07 | 64.40 | - | - | - | - | - | - |
| Morocco | - | - | - |  |  | - | - | - | - | - | - |
| Namibia | - | - | - |  |  | - | - | - | - | - | - |
| Nigeria | - | - | - | 13.97 ± 11.05 | 8.45 ± 7.85 | - | - | - | - | - | - |
| Sao Tome and Principle | - | - | - | 0.00 | 0.00 | - | - | - | - | - | - |
| Senegal | 0.79 ± 0.66 |  | 21.60 ± 1.13 |  | 21.90 |  |  |  |  |  |  |
| South Africa |  | 51.85 ± 55.37 |  | 14.85 ± 17.68 | 24.00 ± 24.04 | 18.38 ± 19.26 | 13.40 ± 14.71 | 6.00 |  |  |  |
| Tanzania | 17.48 ± 29.30 | 1.00 | 1.90 ± 1.70 |  |  | 27.30 | 22.50 | 24.50 |  |  |  |
| Togo |  |  |  |  |  |  |  |  |  |  |  |
| Tunisia |  | 5.80 ± 8.20 |  |  |  | 11.60 | 0.00 |  |  |  |  |
| Uganda |  | 3.40 |  | 5.50 |  |  |  |  |  |  |  |
| Zambia |  | 7.70 | 47.62 |  |  |  | 7.80 | 7.50 |  |  | 10.00 |

SD: Standard deviation; Domestic represent the aggregate average prevalence across the domestic animals included in the analysis

Variability could not be calculated when only one study was available (n = 1); these cases have no SD.

Country-specific molecular prevalence estimates of *Coxiella burnetii* in humans, domestic animals, wildlife, arthropods, and selected animal species. Values are presented as mean prevalence (%) ± standard deviation (SD) where multiple studies were available. Domestic represents the aggregate prevalence across domestic animal species included in the analysis. Variability could not be calculated when only one study was available (n = 1).

**Table S 2. Seroprevalence of Q fever recorded by studies across Africa**

| Country | Human  Mean ± SD | Domestic  Mean ± SD | Wildlife  Mean ± SD | Cattle  Mean ± SD | Sheep  Mean ± SD | Goat  Mean ± SD | Camel  Mean ± SD | Buffalo  Mean ± SD |
| --- | --- | --- | --- | --- | --- | --- | --- | --- |
| Algeria | 7.93 ± 6.55 | 33.63 ± 30.42 | 8.73 | 18.93 ± 15.72 | 16.50 ± 7.28 | 11.87 ± 4.43 | 51.95 ± 33.31 | - |
| Cameroon | - | 34.06 ± 47.99 | - | 34.06 ± 47.99 | - | - | 2.35 | - |
| Central African Republic | - | 14.30 | - | - | - | - | - | - |
| Chad | 49.10 | 10.30 ± 3.54 | - | 7.45 ± 0.49 | 19.10 | 17.10 | 3.10 | - |
| Côte d'Ivoire | - | - | - | 13.90 | 9.40 | 12.40 | - | - |
| Egypt | 33.04 ± 18.80 | 23.29 ± 7.00 | - | 17.43 ± 3.67 | 14.77 ± 11.38 | 21.00 ± 12.30 | 40.70 | 11.20 |
| Ethiopia | 25.65 ± 0.92 | 23.09 ± 13.88 | - | 13.50 ± 10.80 | 28.92 ± 8.21 | 39.69 ± 13.88 | 33.46 ± 20.18 | - |
| Gabon | - | - | - | - | - | - | - | - |
| Gambia | 3.80 | 23.25 ± 2.33 | - | - | 20.40 ± 2.69 | 24.85 ± 0.92 | - | - |
| Ghana | 11.85 ± 2.38 | 21.95 ± 0.49 | - | 21.70 | 22.48 ± 8.37 | 19.29 ± 13.13 | - | - |
| Guinea |  | 9.10 | - | 20.50 | 2.30 | 4.40 | - | - |
| Kenya | 20.93 ± 10.47 | 18.62 ± 17.83 | 10.00 | 9.50 ± 9.70 | 11.81 ± 6.25 | 18.44 ± 11.88 | 29.73 ± 15.47 | - |
| Madagascar | - | 2.00 ± 0.00 | - | 2.00 ± 0.00 | - | - | - | - |
| Mali | - | - | - | 55.30 | 22.60 | 16.90 | 75.54 | - |
| Morocco | - | - | 47.32 | - | - | - | - | - |
| Namibia | 26.10 | 17.60 | - | - | 17.60 | - | - | - |
| Nigeria | 62.57 | 13.50 ± 10.24 | - | 10.93 ± 8.92 | 3.85 | 5.98 ± 3.99 | - | - |
| Sao Tome and Principle | 6.70 | - | - | - | - | - | - | - |
| Senegal | - | - | - | - | - | - | - | - |
| South Africa | 19.77 ± 15.20 | 16.59 ± 14.20 | 26.95 ± 38.11 | 19.76 ± 14.73 | 7.07 ± 6.97 | 9.20 ± 13.01 | 2.73 ± 0.53 | - |
| Tanzania | 3.88 ± 1.58 | 3.86 | - | 1.93 ± 2.73 | 2.30 | 5.00 | - | - |
| Togo | 35.97 | 14.90 | - | 14.80 | 14.40 | 8.30 | - | - |
| Tunisia | - | 41.64 ± 33.21 | - | - | 7.31 | - | 39.98 ± 35.79 | - |
| Uganda | - | - | - | - | - | - | - | - |
| Zambia | - | 5.90 | - | - | 6.28 | 0.00 | - | - |

Domestic represent the aggregate average prevalence across the domestic animals included in the analysis

Variability could not be calculated when only one study was available (n = 1); these cases have no SD.

Country-specific seroprevalence estimates of Coxiella burnetii in humans, domestic animals, wildlife, and selected animal species. Values are presented as mean prevalence (%) ± standard deviation (SD) where multiple studies were available. Domestic represents the aggregate prevalence across domestic animal species included in the analysis. Variability could not be calculated when only one study was available (n = 1).

**Table S 3. Assessing statistical heterogeneity among studies using Cochran’s Q test, I² statistics, and Tau²**

| Variable | Mean | SD | Cv | Q | Q_p-value | I^2^ | Tau^2^ |
| --- | --- | --- | --- | --- | --- | --- | --- |
| arthropod_total | 1450.61111 | 2429.27764 | 167.465809 | 1260 | 4.597E-242 | 97.2222222 | 5737462.35 |
| domestic_total | 749.807229 | 1207.65889 | 161.062583 | 6806 | 0 | 98.7951807 | 1440868.42 |
| human_total | 504.020833 | 453.576402 | 89.991598 | 2256 | 0 | 97.9166667 | 201445.479 |
| mp_arthropods | 11.1153929 | 14.8507049 | 133.604859 | 756 | 6.867E-142 | 96.4285714 | 212.666885 |
| mp_camel | 8.06666667 | 11.1576581 | 138.318075 | 6 | 0.04978707 | 66.6666667 | 82.9955556 |
| mp_cattle | 8.201 | 9.73739693 | 118.734263 | 90 | 1.6281E-15 | 90 | 85.335209 |
| mp_domestics | 12.4835294 | 21.4927092 | 172.168531 | 272 | 1.5526E-48 | 94.1176471 | 434.763811 |
| mp_goat | 7.28333333 | 8.84384909 | 121.425846 | 30 | 1.4749E-05 | 83.3333333 | 65.1780556 |
| mp_humans | 5.8553375 | 12.9511622 | 221.185579 | 240 | 1.4164E-42 | 93.75 | 157.249314 |
| mp_pets | 5.76666667 | 5.17332904 | 89.7109082 | 6 | 0.04978707 | 66.6666667 | 17.8422222 |
| mp_sheep | 9.88111111 | 11.9546816 | 120.985195 | 72 | 1.9625E-12 | 88.8888889 | 127.035032 |
| mp_ticks | 11.6978947 | 16.2452515 | 138.873292 | 342 | 1.0346E-61 | 94.7368421 | 250.01829 |
| mp_wildlife | 18.924 | 18.8518933 | 99.6189668 | 20 | 0.0004994 | 80 | 284.315104 |
| sp_camel | 31.6816667 | 26.1412154 | 82.5121219 | 306 | 6.4955E-55 | 94.4444444 | 645.398525 |
| sp_cattle | 15.126439 | 14.8078225 | 97.8936449 | 1640 | 0 | 97.5609756 | 213.92352 |
| sp_domestic | 21.478963 | 19.4485678 | 90.5470521 | 2862 | 0 | 98.1481481 | 371.242218 |
| sp_goat | 17.944069 | 13.5659016 | 75.6010338 | 812 | 6.414E-153 | 96.5517241 | 177.687698 |
| sp_human | 21.3019231 | 16.3908155 | 76.9452383 | 650 | 4.172E-121 | 96.1538462 | 258.3258 |
| sp_sheep | 15.0119091 | 9.54354083 | 63.5731323 | 1056 | 2.679E-201 | 96.969697 | 88.3191966 |
| sp_wildlife | 23.99 | 24.713381 | 103.015344 | 20 | 0.0004994 | 80 | 488.60096 |
| wildlife_total | 415.357143 | 977.985418 | 235.456507 | 182 | 6.6293E-32 | 92.8571429 | 888137.23 |

sp: seroprevalence; mp: molecular prevalence; domestic: this represent the aggregate average prevalence across the domestic animals included in the analysis; arthropod: this represent the aggregate average prevalence across the domestic animals included in the analysis; arthropod.SD: standard deviation; Cv: coefficient of variation; Q: Cochran’s Q statistic; Q p-value; I^2^: I-squared statistic; Tau^2^: Tau-squared statistic.

Measures of heterogeneity for molecular and serological prevalence estimates across host groups and species. Heterogeneity was assessed using Cochran’s Q statistic, Q p-value, I² statistic, and Tau². Mean prevalence, standard deviation (SD), and coefficient of variation (CV) are also presented. Domestic represents aggregate prevalence across domestic animal species, while arthropod represents aggregate prevalence across arthropod vectors included in the analysis
